# West Nile Virus Retinopathy and Associations with Long Term Neurological and Neurocognitive Sequelae

**DOI:** 10.1371/journal.pone.0148898

**Published:** 2016-03-07

**Authors:** Rodrigo Hasbun, Melissa N. Garcia, Judianne Kellaway, Laura Baker, Lucrecia Salazar, Steven Paul Woods, Kristy O. Murray

**Affiliations:** 1 University of Texas Health Science Center at Houston, Medical School, Section of Infectious Diseases, Houston, Texas, United States of America; 2 Baylor College of Medicine, Department of Pediatrics, Section of Pediatric Tropical Medicine, National School of Tropical Medicine, Houston, Texas, United States of America; 3 University of Texas Health Science Center at Houston, Medical School, Ruiz Department of Ophthalmology and Visual Science, Houston, Texas, United States of America; 4 Robert Cizik Eye Clinic, Houston, Texas, United States of America; 5 Department of Psychology, University of Houston, Houston, Texas, United States of America; University of Texas Medical Branch, UNITED STATES

## Abstract

West Nile virus (WNV) has emerged as an important vector-borne pathogen in North America, with more than 3 million estimated to have been infected. Retinopathy from WNV infection has been previously reported in acute cases, though those prior reports did not evaluate the risk of retinopathy based on clinical severity of neurologic disease. The purpose of this cross-sectional study was to perform comprehensive ophthalmological and neurological examinations on 111 patients with a history of West Nile virus infection and describe the ocular manifestations. Out of 111 patients, 27 (24%) had evidence for West Nile virus associated retinopathy (WNVR); this observation was higher (49%) in those patients who initially presented with encephalitis. Individuals with WNVR had more frequent involvement of the macula and peripheral involvement compared to those patients without WNVR (p<0.05). WNVR was also associated with a greater likelihood of abnormal reflexes on neurological exam, poorer learning, greater dependence in activities of daily living, and lower quality of life (p<0.05). WNVR was seen more frequently in elderly patients (age > 60 years), and was associated with higher rates of diabetes mellitus and a history of encephalitis (p<0.05). A multivariable logistic regression revealed that only a history of encephalitis was independently associated with WNVR [Adjusted Odds Ratio = 4.9 (1.8–13.2); p = 0.001]. Our study found that WNVR occurs in one fourth of patients with a history of WNV infection and is more frequently observed in those with apparent severe neurological sequelae (e.g., encephalitis). The clinical relevance of WNVR was supported by its associations with dependence in activities of daily living and lower quality of life. This unique evaluation of WNV patients included fundoscopic examinations and their associations with neurologic impairment. Our findings can be used during ophthalmological consultation for the evaluation, treatment and rehabilitation phases of care for WNV patients.

## Introduction

West Nile virus (WNV) has infected approximately 3 million adults across the United States since it was first identified in New York City in 1999.[1, 2] WNV is transmitted to humans through the bite of an infected mosquito. Approximately 1% of infected individuals will develop neuroinvasive disease characterized by either meningitis, encephalitis, and/or acute flaccid paralysis.[3] West Nile virus-associated retinopathy (WNVR) has been documented in patients presenting with acute neuroinvasive disease and has been characterized by a multifocal chorioretinitis with or without vitreous inflammatory changes.[4, 5] Linear clustering of chorioretinal scars, sometimes following the course of retinal nerve fibers, is a typical characteristic of WNVR.[1] Other findings described in WNV infections include uveitis, retinitis, retinal hemorrhages, vascular sheathing and leakage, macular edema, vasculitis, optic disc swelling, and retinal pigment epithelium (RPE) changes.[1–6] The objective of our study was to characterize the prevalence of WNVR among a large cohort of patients with a history of WNV infection and explore the association of WNVR to neurological and everyday living implications.

## Methods

A cohort of WNV-positive patients was established in 2002 in Houston, Texas and followed prospectively from the time of acute infection to time of evaluation with a median of 6.8 years (0.1–11 years) post-infection. Study patients were originally identified through local health department surveillance or routine screening of the blood supply at local blood donation centers during 2002–2012. Detailed procedures for diagnosis and confirmation of WNV-positive status were previously described.[7–10] The University of Texas Health Science Center and the Baylor College of Medicine institutional review boards approved our study protocol and procedures. To date, 220 patients have been consented and enrolled into the cohort. From the original cohort, 61 were excluded from this study based on the following criteria: 24 being deceased, 11 determined a false positive case for WNV, 24 were lost-to-follow-up, and/or 6 refusing to continue long-term participation. Of the 159 patients eligible for both ophthalmic and neurologic examinations, 111 (70%) consented and were available to participate. Our final study population included: 26 asymptomatic cases, 36 uncomplicated fever cases, 14 aseptic meningitis cases, and 35 encephalitis cases.

**Fig 1:**
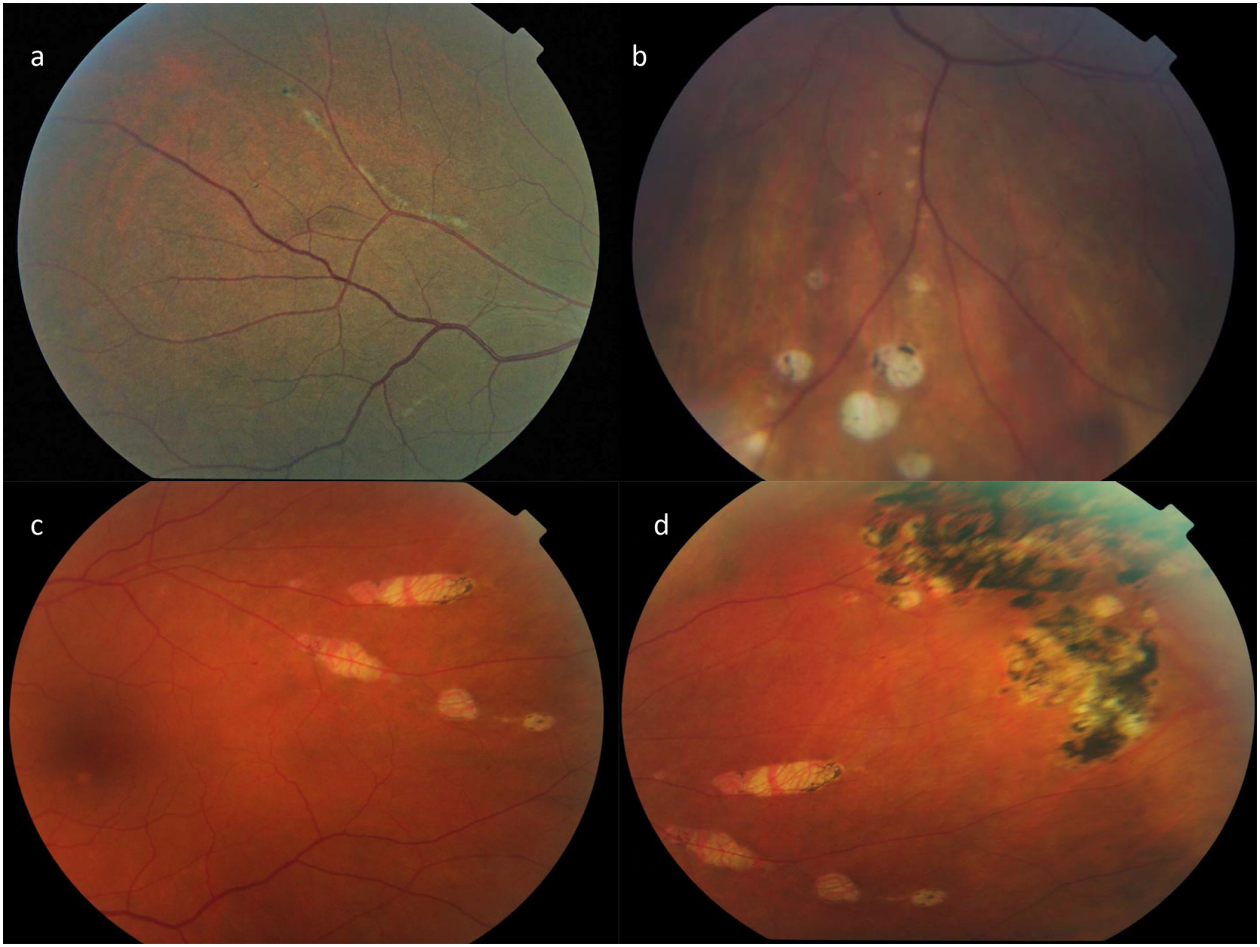
Severe retinopathy seen in four patients with history of West Nile virus infection.

The patients underwent a thorough eye examination which included a history, present and past ocular health, medical and surgical history, and review of systems. Historical information collected from these patients included hospitalization for encephalitis and meningitis, febrile illness, and for some asymptomatic patients, notification by the blood donor agency of their positive result for WNV viremia. Visual acuity with present correction or pinhole was performed using a Snellen chart. Intraocular pressures were performed in those patients who agreed to have it done. The anterior segment was examined with biomicroscopy, and a dilated fundus examination was performed. Fundus photography with a Topcon camera was performed to document abnormal findings. The main outcome of the study was the presence of WNVR that was defined as a characteristic multifocal chorioretinitis with linear clustering of chorioretinal scars that was easily differentiated from diabetic or hypertensive retinopathy, or other ophthalmic disease (see Fig 1).[1, 3, 4] Any clinical finding that could not be definitively characterized as WNVR as previously reported in the literature was not included.

A complete neurological exam was performed and included testing of cranial nerves, strengths of all muscle groups, tone, sensory examination, deep tendon reflexes, assessment for tremors and cerebellar examination. The presence of any abnormality in the neurological exam was categorized as an abnormal neuro examination. The Barthel Index assessed the ability to perform activities of daily living,[11] the SF-36 was used to measure quality of life,[12] the Beck Depression Inventory was used to assess recent mood,[13] and the Modified Fatigue Impact Scale (MFIS) measured the impact of fatigue on physical, cognitive and psychosocial functioning.[14] The Repeatable Battery for Assessment of Neuropsychological Status (RBANS) test was administered to provide a comprehensive screening of relevant neurocognitive functions,[15] including attention, language, visuospatial construction abilities, and immediate and delayed memory.

The *t*-test was used to analyze differences in baseline clinical characteristics, neurological exam and ophthalmological exam findings between patients with and without WNVR. To assess differences in the RBANS, Barthel index, SF-36, Modified Impact Fatigue Scale and Beck Depression Inventory, test scores were reported as mean and standard deviation, and an F value and p value were calculated by the a one-way analysis of variance. A p value <0.05 was considered significantly different between both groups. Bivariate analysis were then conducted by using the Pearson’s X^2^ test or Fisher’s exact test to identify factors that were significantly associated with WNVR (P < 0.05). Only clinically cogent baseline variables showing a bivariate association (P<0.05) were entered into a step-wise logistic regression model to verify independent associations with an adverse clinical outcome. Bootstrap analysis was performed to validate the model internally. All analyses were performed by IBM^®^ SPSS^®^ version 21.

## Results

Among a cohort of patients with a history of WNV infection, 24% (27/111) had evidence of WNVR. Of the 35 patients with an encephalitis presentation, 17 (49%) had evidence of WNVR, compared to none of the 14 meningitis cases, 9 (25%) of the 36 uncomplicated fever cases, and 1 (4%) of the 26 asymptomatic cases.

In the 27 patients with ocular findings of WNVR, the visual acuity ranged from 20/20 to 20/70. The anterior segment was carefully examined for evidence of previous or current inflammation such as the presence of cell and/or flare, posterior synechiae, fibrin, iris atrophy and pigment on the anterior lens capsule. One patient showed iris synechiae indicative of previous anterior uveitic disease, which may have been related to WNV. No other patients had anterior segment evidence of old (or current) inflammatory or infectious disease. Incidental findings considered not to be related to WNV in the anterior segment which are consistent with the age of this population included dermatochalasis, ptosis, corneal scars, guttata, cataracts and pseudophakia. A few patients had significant anatomical narrowing of the anterior chamber angle. These patients were not dilated, and were referred to the appropriate specialist for evaluation of angle closure glaucoma.

The fundoscopic examination in these patients revealed a variety of clinical findings, some considered to be related to previous WNVR infections, and other findings considered incidental or unrelated to previous WNVR. Findings included the classic curvilinear pattern of chorioretinal scarring, other chorioretinal scarring occurring in clusters, optic nerve pallor, peripapillary sheathing, and vascular sheathing. In one patient an epiretinal membrane in the macula was consistent with other findings of previous WNV infection (optic nerve pallor and multiple chorioretinal scars).

Patients with WNVR were older, had higher prevalence of coexisting diabetes mellitus, and were more likely to have had an encephalitis presentation (p<0.05) (see Table 1). There were no significant differences seen regarding race, gender, or education level. An abnormal neurological exam was present in 17 (62%) of the patients with WNVR. Abnormal deep tendon reflexes were seen more commonly in the WNVR group (7/27 (25.9%) vs. 7/84 (8.3%); P = 0.039). Patients with WNVR also had other neurological abnormalities of the spinal cord identified on exam such as motor weakness (29.6%), and sensory deficits (7.4%) but their incidence was not significantly different from those WNV patients without retinopathy.

**Table 1:** Baseline Characteristics, coexisting medical conditions and neurological findings in patients with West Nile virus infections.

Variable, n (%)	No WNV retinopathy (n = 84)	WNV retinopathy (n = 27)	P Value ^a^
Median age, (range)	57 years (18–89)	66 (26.4–86.3)	**0.02**
Male sex	46/84 (55)	14/27 (52)	0.82
White race	70/84 (83)	23/27 (85)	1.00
Education level >12 years	63/84 (75)	15/27 (56)	0.09
Hypertension	24/80 (30)	12/26 (46)	0.16
Diabetes mellitus	7/83 (8)	8/27 (30)	0.01
Encephalitis presentation^b^	18/84 (21)	17/27 (49)	<0.0001
Abnormal neurological exam^c^	36/84 (43)	17/27 (63)	0.08
Abnormal strength	19/84 (23)	8/27 (30)	0.45
Abnormal reflexes	7/84 (8)	7/27 (26)	0.04
Tremors	8/84 (10)	3/27 (11)	0.73
Abnormal sensory exam	4/84 (5)	2/27 (7)	0.63
Abnormal cerebellar exam	1/84 (1)	1/27 (4)	0.43

^a^ P value comparing patients with and without West Nile virus retinopathy, P <0.05 considered significant

^b^ initial presentation with altered mental status, focal neurological signs or seizures

^c^ any abnormality on a comprehensive neurological exam

Patients with evidence of previous WNVR were also more likely to have other abnormalities in the macula, iris, and in the periphery of the retina (P<0.01) (see Table 2). Macular and peripheral abnormalities seen on exam included retinal pigment epithelium (RPE) mottling in the papillomacular bundle as well as other areas of the macula, drusen, cotton wool spots, microaneurysms, hemorrhages, epiretinal membrane macular degeneration, geographic atrophy, cystic retinal tuft, peripheral RPE mottling, pigmented and hypopigmented chorioretinal scars of varying sizes, choroidal nevus, paving stone degeneration, and pigmented lattice.

**Table 2:** Ophthalmological findings in 111 patients with West Nile virus infection.

Abnormal eye findings	No WNV retinopathy (n = 84)	WNV retinopathy (n = 27)	P Value ^a^
Visual field OD	3/84 (3.6)	2/27 (7.4)	0.63
Visual field OS	3/84 (3.6)	1/27 (3.7)	0.84
Visual acuity OD ^b^	11/65 (16.9)	1/23 (4.3)	0.13
Visual acuity OS ^b^	10/80 (12.5)	4/25 (16)	0.65
Eye lid OD ^c^	12/84 (14.3)	7/27 (2.6)	0.16
Eye lid OS ^c^	11/84 (13.1)	9/27 (33.3)	0.02
Conjunctiva OD ^d^	20/84 (23.8)	10/27 (37.0)	0.18
Conjunctiva OS ^d^	19/84 (22.6)	10/27 (37.0)	0.14
Pupil OD ^e^	2/84 (2.4)	1/27 (3.7)	0.57
Pupil OS ^e^	1/84 (1.2)	1/27 (3.7)	0.43
Lens OD ^f^	57/84 (67.8)	19/27 (70.3)	0.81
Lens OS ^f^	55/84 (65.5)	19/27 (70.3)	0.64
Cornea OD ^g^	16/84 (19.0)	4/27 (14.8)	0.78
Cornea OS ^g^	20/84 (23.8)	3/27 (11.1)	0.18
Anterior chamber OD ^h^	7/84 (8.3)	4/27 (14.8)	0.46
Anterior chamber OS ^h^	8/84 (9.5)	4/27 (14.8)	0.48
Iris OD ^i^	0/84 (0)	3/27 (11.1)	0.01
Iris OS ^i^	0/84 (0)	4/27 (14.8)	0.003
Optic disc OD ^j^	4/78 (5.1)	4/27 (14.8)	0.20
Optic disc OS^j^	2/77 (2.6)	6/27 (22.2)	0.003
Vitreous OD ^k^	1/78 (1.3)	0/27 (0)	1.0
Vitreous OS ^k^	1/78 (1.3)	0/27 (0)	1.0
Macula OD ^l^	9/78 (11.5)	14/27 (51.8)	<0.001
Macula OS ^l^	10/78 (12.8)	11/27 (40.7)	0.002
Vessel OD ^m^	2/78 (2.6)	2/27 (7.4)	0.27
Vessel OS ^m^	2/78 (2.6)	3/27 (11.1)	0.11
Periphery OD ^n^	11/78 (14.1)	20/27 (74.1)	<0.001
Periphery OS ^n^	10/78 (12.8)	20/27 (74.1)	<0.001

^a^ P value <0.05 considered significant; Fisher Exact used when cell count was less than 5.

^b^ abnormal visual acuity defined as >20:40

^c^ eye lid abnormalities included blepharitis, dermatitis, lid laxity, ptosis, hyperemia, scurf, trichiasis, injection, dermatochalasis

^d^ conjunctival abnormalities included hyperemia, injection, dilated vessels, pinguecula, cysts, subconjuctival hemorrhage, follicular response, scarring, lymphagiectasias

^e^ pupillary abnormalities included persistent pupillary membrane, abnormal dilation and reactivity, tonic pupil and irregularity due to synechia

^f^ cataracts, pigmentation of anterior capsule, nuclear sclerosis, vacuoles, posterior capsule intraocular lens

^g^ radial keratotomy and astigmatic keratotomy scars, arcus, pigmentation, LASIK(Laser-Assisted in situ Keratomileusis) flap scars, dry eye, pannus, keratopathy, guttata, microcysts, pterygium, keratoconus,

^h^ persistent pupillary membrane, cells, flare, shallow peripherally or temporally, narrow peripherally, posterior embryotoxin, shallow angles.

^i^ nevi, synechia, wrinkles, brown, bowed peripherally, ectropion,

^j^ cup to disc ratio >0.5, scleral crescent, pigmented crescent, sheathing, peripapillary atrophy, spontaneous venous pulsation, pallor.

^k^ posterior vitreous detachment, floaters, Weiss ring, syneresis, asteroid hyalosis

^l^ disc area mottling, drusen, blond fundus, cotton wool spots, microaneurysms, hemorrhages, cystic retinal tuft, dull fovea reflex, epiretinal membrane, mottling, hypopigmented areas, hypopigmented scars, chorioretinal scars, macular degeneration, microaneurysm, fovea, pigment mottling, retinal pigment epithelium, geographic atrophy, nevus

^m^ arteriovenous nicking, venous enlargement, nevus across superior arcades, tortous vessels, blockages, attenuation, scars

^n^ paving stone degeneration, punched out lesion, pigmented spot, cotton wool spots, microaneurysms, chorioretinal scars, dot blot hemorrhages, drusen, nevus, pigmented lattice, chorioretinal degeneration, mottling, retinal pigment epithelium changes, hypertensive retinopathy

Clinical findings in the iris recorded in this WNVR group included iris nevi, and ectropion uvea. Abnormalities in the optic disc and eyelid were seen more frequently in WNVR but in only one eye (OS) (P<0.05). The abnormalities of the optic disc included a cup to disc ratio >0.5, scleral crescents, sheathing, peripapillary atrophy, spontaneous venous pulsation, pallor. Eyelid findings in this group included blepharitis, dermatochalasis, dermatitis, tattoo, lid laxity, ptosis, hyperemia, trichiasis. There were no significant differences between those with and without WNVR regarding visual fields, visual acuity, conjunctiva, pupils, lens, cornea, anterior chamber, vitreous fluid, and vessels. It should be noted that many of these clinical findings are not specifically related to previous WNVR and simply demonstrate a detailed characterization of the eye exam of these patients

WNVR was associated with significantly lower RBANS scores in the domain of immediate memory (F = 5.1, p = 0.02) (see Table 3). There were no statistically significant differences in visuospatial construction abilities, language, attention, or delayed memory (p > 0.1). WNVR were more likely to report lower quality of life on the SF-36 (F = 9.5, p = .003) and experience difficulties in the performance on activities of daily living as assessed by the Barthel index (F = 6.7, P = 0.01). There were no significant differences observed in chronic fatigue or depression between those with and without WNVR (p>0.2).

**Table 3:** Neurocognition, fatigue, depression and activities of daily living in WNV retinopathy.

Test Scores^a^	No WNV retinopathy (n = 84)	WNV retinopathy (n = 27)	F value/p-value ^b^
**RBANS**^c^**—Immediate memory**	94.4 (17.5)	85.6 (17.7)	5.1/0.02
**RBANS**^c^**—Visuospatial construction**	106.8 (15.9)	102.0 (17.2)	1.4/0.24
**RBANS**^c^**—Language**	96.2 (11.0)	95.1 (9.0)	0.2/0.64
**RBANS**^c^**—Attention**	103.4 (18.9)	101.1 (21.9)	0.27/0.60
**RBANS**^c^**—Delayed memory**	96.5 (17.4)	93.6 (18.4)	0.54/0.46
**Barthel index** ^d^	99.3 (2.4)	96.3 (10.0)	6.7/0.01
**Modified fatigue impact scale (MFIS)** ^e^	24.9 (22.2)	28.9 (23.5)	0.54/0.46
**Beck’s depression inventory** ^f^	9.0 (10.5)	9.9 (9.9)	0.15/0.69
**SF-36**	100.2 (8.3)	93.2 (14.8)	9.5/0.003

^a^ test scores are reported as mean and standard deviation (in parentheses).

^b^ F value and p value as calculated by the analysis of variance. A p value <0.05 was considered significantly different between both groups.

^c^ Repeatable Battery for the assessment of neuropsychological status. RBANS scores are age- and education-corrected standardized score to a median of 100.

^d^ Barthel index is an ordinal scale that measures activities of daily living.

^e^ MFIS is an instrument that provides an assessment of the effects of fatigue in terms of physical, cognitive, and psychosocial functioning.

^f^ 21-question multiple-choice self-report inventory to assess presence and severity of depression

On bivariate analyses, baseline clinical factors associated with WNVR included age >60 years, diabetes mellitus, and encephalitis presentation (P<0.05). (See Table 4). An abnormal neurological exam was not statistically associated with WNVR. All significant variables on bivariate analyses were entered into a multivariable logistic regression analyses, and only an encephalitis presentation remained associated with WNVR [Adjusted OR 4.9 (1.8–13.2) P = 0.001].

**Table 4:** Bivariate analyses and Logistic Regression Analysis of Factors Associated with WNV Retinopathy.

Characteristics	Bivariate		Logistic regression	
	Odds Ratios (95% CI)^a^	P-value	Odds Ratios (95% CI)	P-value
**Encephalitis**^b^	6.2 (2.4–15.9)	<0.001	4.9 (1.8–13.2) ^c^	0.001
**Diabetes mellitus**	4.5 (1.5–14.2)	0.008	2.7 (0.8–9.7)	0.12
**Age > 60 years**	2.4 (0.97–5.8)	0.057	1.5 (0.5–4.0)	0.47
**Abnormal neurological exam**^d^	2.3 (0.9–5.5)	0.07	2.0 (0.7–5.4)	0.17

Footnotes:

^a^ 95% confidence intervals,

^b^ clinical presentation with altered mental status, seizures or focal neurological abnormalities,

^c^ validated by bootstrap analysis (p = 0.001)

^d^any abnormality on a comprehensive neurological exam

## Discussion

Our study of retinopathy among patients with a history of WNV infection has many important and unique findings. To our knowledge, this is the largest study to assess evidence of retinopathy in a WNV cohort that included asymptomatic, febrile, and neuroinvasive disease patients. Additionally, this is the first study correlating WNVR with neurological exam abnormalities, neurocognition, depression, fatigue, and with activities of daily living. We found that 24% of the cohort had evidence of WNV-related retinopathy. This retinopathy was seen in 49% of patients with WNV infection that presented with encephalitis and had a distinctive multifocal chorioretinitis characterized by linear clustering of hypopigmented scars. Interestingly, we also observed some subtle retinal pigment epithelium (RPE) mottling in several patients, which has not been reported in WNVR. It is possible that this RPE mottling represents an abnormality from previous WNVR, and these findings should be studied further in the future. (See Fig 1)

As to be expected, we observed other posterior segment findings that were clearly unrelated to WNV infection. Some patients exhibited macular or peripheral drusen, one patient had a retinal hemorrhage related to macular degeneration, and one patient had diabetic retinopathy. All of these disease entities are distinctly different in their appearance, well known to a general ophthalmologist, and unrelated to WNV infection. No active vitritis was found in any of our patients, and no vitreous cells were found in any of the patients, which would indicate prior inflammatory disease. Occasionally a small, isolated darkly pigmented lesion or atrophic spot were seen and could not definitively be attributed to WNV, but rather could be an incidental finding unrelated to the WNV infection. Possibilities include old chorioretinal scars due to reactive hyperplasia of the RPE, presumed ocular histoplasmosis, or other injuries, infections or inflammatory entities; however, the association with WNV cannot be excluded. These patients were not included in the WNVR group for analysis of the data.

WNV is a positive-sense RNA virus from the Flaviviridae family that has a distinctive neurotropism.[16] Following infection, WNV patients continue to experience chronic neurological abnormalities including cognitive impairment, depression and fatigue.[14, 17, 18] In our cohort study, 55 (48%) had an abnormal neurological examination. Abnormal deep tendon reflexes were the only neurological abnormality seen more commonly in the WNVR group (7/27 (26%) vs. 7/84 (8.3%) (p = 0.039). WNV can cause acute flaccid paralysis due to involvement of the anterior horn cells, and motor polyneuropathies [19] that could explain some of the abnormal deep tendon reflexes found in our study population.

Patients with WNVR were more likely to have abnormalities in the macula (e.g., epiretinal membrane, scars) and in the periphery of the retina (p<0.01) (see Table 2). WNV is known to cause a multifocal chorioretinitis that involves both the macula and the periphery of the retina and can be associated with vitreous inflammation.[1, 3, 4, 20] Macular involvement is important to recognize early as it can lead to central visual impairment. Intravitreal bevacizumab has been used successfully to treat macular edema in a patient with acute WNV infection.[21] Multifocal chorioretinitis was present in 27 out of 36 (75%) of patients with WNV and was the only predictor in logistic regression analyses to be association with WNVR.[20] In our study, we also observed a similar rate of multifocal chorioretinitis in 20 out of 27 (74%) with WNVR. A prospective study of 38 patients with WNV infection identified diabetes mellitus as an independent predictor of having WNVR.[22] Within our cohort that included a much larger sample size, WNVR was associated with encephalitis, diabetes mellitus, and age greater than 60 years of age in bivariate analysis, but only encephalitis was associated with WNVR on logistic regression analyses.

Patients with WNVR were more likely to have lower scores on a well-validated measure of immediate memory (i.e., story and complex figure learning). At the clinical level, WNVR was associated with notable problems in everyday functioning, including dependence activities of daily living as documented by the Barthel index and lower general quality of life. Patients with WNVR and neurological sequelae show significant changes in their activities of daily living. Our finding of neurological impairment in those with WNVR is most likely due to the high prevalence of WNVR in those who had an acute clinical presentation of encephalitis. Almost half (49%) of our patients who experienced encephalitis had evidence of WNVR, and this was found to be highly statistically significant (p<0.001) when compared to less severe clinical presentations. We recently conducted a study to examine recovery over time from WNV infection and found those with encephalitis were significantly more likely to have poorer recovery, with 70% reporting continued disability more than 5.5 years following infection (MURRAY PLoS ONE ref). Interestingly, in that study we also found those with a presentation of uncomplicated fever had poorer recovery when compared to meningitis cases, with 60% still reporting symptoms more than 5.5 years following infection. In this current study, one quarter of uncomplicated fever cases had evidence of WNVR compared to 0 meningitis cases and 1 (4%) asymptomatic case. As we speculated in the prior study, it is possible that these presumed uncomplicated fever cases are misclassified since most were hospitalized for their infection but did not have a lumbar puncture procedure to confirm neuroinvasive disease. These findings warrant further investigation.

The ocular findings due to WNV in our cohort are similar to what has been reported in other studies.[1, 3–5] Our study though has several advantages over previous work. First, this is the largest prospective cohort study of WNV infected patients that have undergone a thorough dilated fundoscopic examination documenting the largest series of WNVR. Second, our assessment of the WNV infected patients include a comprehensive neurological assessment that included a complete neurological exam, neurocognitive evaluation and assessment for depression, fatigue and impact in activities of daily living. Third, in order to reduce the hazards of multiple statistical contrasts, bivariate analysis emphasized only clinically plausible variables and the inclusion of limited number of variables allowed us to avoid hazards of overfitting in multivariable analysis.[23]

Despite its methodological advances, our study had limitations. First, our study lacked age-matched controls to compare patients without WNV infection in order to assess other causes of eye abnormalities. Second, we did not have a baseline examination before the onset of the WNV infection for comparison. Third, there is the possibility of misclassification of some of the West Nile encephalitis cases as not all patients with WNV infection underwent a lumbar puncture at time of diagnosis. Finally, we had a large number of deaths (n = 24) among our patient population, and deaths occurred mostly in individuals who had neuroinvasive disease. Unfortunately, we were unable to perform ophthalmic exams prior to their death, and excluding their findings could create some bias to our analysis.

In summary, WNVR is seen in almost half of patients with WNV encephalitis and is associated with neurological abnormalities, impaired memory and has an impact of activities of daily living. Routine ophthalmological consultation should be considered in patients presenting with WNV encephalitis as retinopathy can be associated with visual loss. It is feasible, for example, that steroid or anti-VEGF therapies for inflammation or edema in the acute care setting might help decrease vision loss in these patients. Many of these patients who present with severe neuroinvasive disease are initially managed in intensive care settings, where the ophthalmological exam might be considered secondary, when in fact, it might identify disease that could be amenable to treatment. Future studies should aim to try to identify the specific mechanisms of ocular involvement and potential therapies during acute WNV disease.

## References

[1] Khairallah M, Ben Yahia S, Attia S, Zaouali S, Ladjimi A, Messaoud R. Linear pattern of West Nile virus-associated chorioretinitis is related to retinal nerve fibres organization. Eye. 2007;21(7):952–5. Epub 2006/04/22. 10.1038/sj.eye.6702355 . 16628235

[2] Shukla J, Saxena D, Rathinam S, Lalitha P, Joseph CR, Sharma S, et al Molecular detection and characterization of West Nile virus associated with multifocal retinitis in patients from southern India. International journal of infectious diseases: IJID: official publication of the International Society for Infectious Diseases. 2012;16(1):e53–9. Epub 2011/11/22. 10.1016/j.ijid.2011.09.020 . 22099888

[3] Khairallah M, Ben Yahia S, Ladjimi A, Zeghidi H, Ben Romdhane F, Besbes L, et al Chorioretinal involvement in patients with West Nile virus infection. Ophthalmology. 2004;111(11):2065–70. Epub 2004/11/04. 10.1016/j.ophtha.2004.03.032 . 15522373

[4] Chan CK, Limstrom SA, Tarasewicz DG, Lin SG. Ocular features of west nile virus infection in North America: a study of 14 eyes. Ophthalmology. 2006;113(9):1539–46. 10.1016/j.ophtha.2006.04.021 . 16860390

[5] Khairallah M, Ben Yahia S, Attia S, Zaouali S, Jelliti B, Jenzri S, et al Indocyanine green angiographic features in multifocal chorioretinitis associated with West Nile virus infection. Retina. 2006;26(3):358–9. Epub 2006/03/02. . 16508441 10.1097/00006982-200603000-00019

[6] Sivakumar RR, Prajna L, Arya LK, Muraly P, Shukla J, Saxena D, et al Molecular diagnosis and ocular imaging of West Nile virus retinitis and neuroretinitis. Ophthalmology. 2013;120(9):1820–6. 10.1016/j.ophtha.2013.02.006 . 23642374

[7] Murray KO, Garcia MN, Rahbar MH, Martinez D, Khuwaja SA, Arafat RR, et al Survival analysis, long-term outcomes, and percentage of recovery up to 8 years post-infection among the Houston West Nile virus cohort. PLoS One. 2014;9(7):e102953 10.1371/journal.pone.0102953 25054656 PMC4108377

[8] Murray KO, Garcia MN, Yan C, Gorchakov R. Persistence of detectable immunoglobulin M antibodies up to 8 years after infection with West Nile virus. The American journal of tropical medicine and hygiene. 2013;89(5):996–1000. 10.4269/ajtmh.13-0232 24062481 PMC3820351

[9] Murray KO, Baraniuk S, Resnick M, Arafat R, Kilborn C, Shallenberger R, et al Clinical investigation of hospitalized human cases of West Nile virus infection in Houston, Texas, 2002–2004. Vector borne and zoonotic diseases. 2008;8(2):167–74. 10.1089/vbz.2007.0109 . 18399781

[10] Murray K, Baraniuk S, Resnick M, Arafat R, Kilborn C, Cain K, et al Risk factors for encephalitis and death from West Nile virus infection. Epidemiology and infection. 2006;134(6):1325–32. 10.1017/S0950268806006339 16672108 PMC2870518

[11] Huybrechts KF, Caro JJ. The Barthel Index and modified Rankin Scale as prognostic tools for long-term outcomes after stroke: a qualitative review of the literature. Current medical research and opinion. 2007;23(7):1627–36. Epub 2007/06/15. 10.1185/030079907X210444 . 17559756

[12] Ramanuj PP, Granerod J, Davies NW, Conti S, Brown DW, Crowcroft NS. Quality of life and associated socio-clinical factors after encephalitis in children and adults in England: a population-based, prospective cohort study. PLoS One. 2014;9(7):e103496 Epub 2014/07/30. 10.1371/journal.pone.0103496 25072738 PMC4114751

[13] Shafer AB. Meta-analysis of the factor structures of four depression questionnaires: Beck, CES-D, Hamilton, and Zung. Journal of clinical psychology. 2006;62(1):123–46. Epub 2005/11/16. 10.1002/jclp.20213 . 16287149

[14] Garcia MN, Hause AM, Walker CM, Orange JS, Hasbun R, Murray KO. Evaluation of prolonged fatigue post-West Nile virus infection and association of fatigue with elevated antiviral and proinflammatory cytokines. Viral immunology. 2014;27(7):327–33. Epub 2014/07/26. 10.1089/vim.2014.0035 25062274 PMC4150370

[15] Lippa SM, Hawes S, Jokic E, Caroselli JS. Sensitivity of the RBANS to acute traumatic brain injury and length of post-traumatic amnesia. Brain injury. 2013;27(6):689–95. Epub 2013/05/16. 10.3109/02699052.2013.771793 . 23672444

[16] Kuno G, Chang GJ, Tsuchiya KR, Karabatsos N, Cropp CB. Phylogeny of the genus Flavivirus. Journal of virology. 1998;72(1):73–83. Epub 1998/01/07. ; PubMed Central PMCID: PMCPmc109351. 9420202 10.1128/jvi.72.1.73-83.1998 PMC109351

[17] Sejvar JJ. The long-term outcomes of human West Nile virus infection. Clinical infectious diseases: an official publication of the Infectious Diseases Society of America. 2007;44(12):1617–24. 10.1086/518281 . 17516407

[18] Nolan MS, Hause AM, Murray KO. Findings of long-term depression up to 8 years post infection from West Nile virus. Journal of clinical psychology. 2012;68(7):801–8. Epub 2013/08/10. 10.1002/jclp.21871 . 23929558 PMC6211791

[19] Leis AA, Stokic DS. Neuromuscular manifestations of west nile virus infection. Frontiers in neurology. 2012;3:37 Epub 2012/03/31. 10.3389/fneur.2012.00037 22461779 PMC3309965

[20] Abroug F, Ouanes-Besbes L, Letaief M, Ben Romdhane F, Khairallah M, Triki H, et al A cluster study of predictors of severe West Nile virus infection. Mayo Clinic proceedings. 2006;81(1):12–6. Epub 2006/01/28. . 16438473 10.4065/81.1.12

[21] Afshar AR, Hariprasad SM, Jampol LM, Sheth VS. Use of intravitreous bevacizumab to treat macular edema in West Nile virus chorioretinitis. Archives of ophthalmology. 2012;130(3):396–8. Epub 2012/03/14. 10.1001/archopthalmol.2011.1630 . 22411674

[22] Khairallah M, Yahia SB, Letaief M, Attia S, Kahloun R, Jelliti B, et al A prospective evaluation of factors associated with chorioretinitis in patients with West Nile virus infection. Ocular immunology and inflammation. 2007;15(6):435–9. Epub 2007/12/19. 10.1080/09273940701798488 . 18085487

[23] Concato J, Feinstein AR, Holford TR. The risk of determining risk with multivariable models. Annals of internal medicine. 1993;118(3):201–10. Epub 1993/02/01. . 8417638 10.7326/0003-4819-118-3-199302010-00009

